# Medical Opinion and Movement

**Published:** 1908-07-11

**Authors:** 


### Medical Opinion and Movement.

DR. HENRY TUCKER, of Philadelphia, contributes to the "Therapeutic Gazette" an account of his experience of magnesium sulphate solution as an empirical local application in cases of erysipelas. The author had previously obtained successful results from the local use of this solution in other varieties of inflammation. The report includes fifty-four severe cases of erysipelas, chiefly affecting the face, which were treated in the Philadelphia General Hospital by the local application of a saturated solution of magnesium sulphate in water. In none of the cases was internal treatment attempted, unless urgently indicated by some complication. Only three of the patients died, and in each instance there were grave complications present. All the other patients recovered completely in from two to seven days. The solution is applied in facial cases on a mask consisting of from fifteen to twenty thicknesses of ordinary gauze, wide enough to extend well beyond the area involved, a small opening being made to permit breathing, but none for the eyes. The saturated mask is applied and covered with oiled silk, and is kept constantly moist. It should not be removed oftener than once in twelve hours to permit an inspection of the parts, and then immediately reapplied; the infected area should not be washed while the treatment is employed. The advantages claimed by the author for this form of treatment are that the drug is accessible, soluble, inexpensive, non-toxic, clean, and easy of application; that the patient obtains prompt relief from distressing local symptoms; that the temperature rapidly falls to normal, usually during the second twenty-four hours, and does not rise again; and that internal medication can be dispensed with in uncomplicated cases, the only treatment being a milk diet until the temperature becomes normal.

THE one great drawback to the efficacy of digitalis when administered by the mouth in cases of failing heart arising from any cause is the fact that it takes three days or so for the full effects of the drug to be felt. Lust, in the *D. Arch f. Klin. Medezin.*, gives a favourable report on the treatment of such cases by a single intravenous injection of strophanthine. The treatment is, moreover, efficacious in such cases as are unsuitable for the exhibition of digitalis, or which resist its effects. Sometimes, however, it is necessary to repeat the injection at the end of a few hours. The first effect noticeable is that the pulse is increased in force and diminished in frequency. The patient experiences a feeling of relief, which is increased when, at the end of a few hours, a copious diuresis sets in. This effect can be maintained by repeating the injection, using a smaller dose of the drug, or by giving digitalis in the ordinary manner. Lust recommends that the drug be injected in doses of 1 milligramme—an aqueous solution may be used and from one-half to one cubic centimetre injected. In grave cases he advises that a preliminary injection of half the usual strength be given, followed later by a second. It is well in cases where the kidneys are acting badly to accompany the injections with diuretics by the mouth. The treatment is of value because its effects are felt so quickly; it is almost a question of minutes only before the patient experiences relief.

IN the *Gaz. Méd. de Nantes*, Dr. V. Tenier discusses the appropriate treatment of children suffering from the effects of foreign bodies lodged in the larynx. He sums up the results of his experience as follows. No expectant treatment is permissible; treatment must be active, and undertaken at once. Emetics or tickling the fauces to induce vomiting must alike be avoided, for they only tend to favour the descent of the foreign body into the bronchi. No attempt should be made to remove the foreign body without the help of a laryngeal mirror; but the child can with advantage be placed with the head low, a position which would naturally favour the expulsion of an object of some little weight. In a few cases the foreign body has escaped spontaneously, when the spasm of the glottis has been overcome by small doses of some anæsthetic. Should these manipulations fail, intervention is necessary. If the object lies in the vestibule of the larynx an attempt should be made to extract it by forceps after cocainisation of the larynx. It is possible by these means to extract foreign bodies situated in the vestibule, between the cords, and even in a lower situation.

OFTEN, unfortunately, this procedure fails, and it is necessary to have recourse to tracheotomy or laryngotomy. If asphyxia is threatening, tracheotomy may be urgently needed, and it will then be necessary to await until later a thorough exploration of the laryngeal region. Up to the age of seven chloroform should be used as an anæsthetic; after that age local anæsthesia, induced by subcutaneous injections of cocaine, or, better still, of alypine may be tried with advantage. The incision should be below the cricoid, through the first three tracheal rings. If there be but slight hæmorrhage, the edges of the wound may be immediately drawn apart by blunt hooks. This may occasionally, if the object lie below the glottis, show the foreign body fixed in the trachea, or the object may present in the wound during inspiration or expiration. If such be the case small forceps will remove the object. Sometimes, however, a thyrotomy is necessary after a preliminary tracheotomy. In such cases the edges of the cartilage should be sewn together immediately the object is removed, unless the subglottic exploration has been difficult, and a great deal of manipulation necessary, in which case the wound should be drained and closed later.

Professor Bach, of Marburg, has some interesting remarks on the Argyll-Robertson pupil in a paper translated by Dr. T. H. Butler. He locates the lesions which cause this phenomenon in the cerebrum or the tracts between it and the lower centres: at least they must be above the nucleus of the third nerve. The pupils are often unequal, and myosis is the rule; but in early cases the size of the pupils may vary from day to day: irregular pupils are common, but by no means characteristic. The phenomenon may be unilateral, or more marked on one side. In typical cases, as is well-known, the direct and consensual light reflexes are not obtained; but the accommodation reflex may be not merely present, but accentuated. The cause of the myosis is very doubtful: it may be due to a failure to dilate to sensory stimuli, and cannot of course be a response to light. A strong protest is made against the assumption that a reactionless pupil is an advanced stage of the Argyll-Robertson: these phenomena are to be kept absolutely distinct, and when one follows the other Bach would ascribe them to separate lesions. Curiously enough Mr. Sydney Stephenson has quite lately described the case of a boy of 13 who had signs of juvenile tabes, and in whom both eyes were reactionless. After two and a half years one pupil was still in this condition, but the other showed the Argyll-Robertson phenomenon. Bach, we gather, would deny the title of Argyll-Robertson to such a recovering inactive pupil; but if so acute an observer as Mr. Stephenson confuses the two, the distinction must be one not easily made.

---

Buchmann, working in Subbotin's clinic at the St. Petersburg Military Medical Academy, has tried the transplantation of articular surfaces, a step in the treatment of articular lesions of which much has lately been heard. His cases were chiefly ankylosed elbows, in which various methods of treatment have been tried, with varying success, of late years. None of these new methods has, however, generally commended itself, and surgeons, on the whole, still hold an elbow-joint ankylosed in a convenient position as far preferable to a weak joint which allows some movement by means of a pseudo-arthrosis obtained by these new methods. The author reports two instructive cases. In one, a fourteen-year-old girl with a completely immovable elbow, the elbow and radio-ulnar joints were completely resected, and an unopened totally resected first metatarso-phalangeal joint was transplanted to the site between humerus and forearm bones. The girl made a good recovery, and is now able to move her arm well, and to bend the forearm almost to a right angle with the upper arm. In the second case a girl of nineteen was operated upon in a similar manner, the result being equally encouraging. The author concludes that transplantation of the whole of a first metatarsal-phalangeal joint is an excellent method of ensuring a movable elbow after total resection of the elbow-joint. It is unnecessary to fix the bones so long as the arm is immobilised in a plaster splint for the first week. Passive movement should be commenced as early as possible.

The clinical picture presented by a typical case of angina pectoris (a disease which it is worth while to note, seems to be common in members of the medical profession) is one that is familiar enough, unfortunately, to most practitioners. The relative impotence of the attendant physician to do more than ameliorate the symptoms of a patient with angina is a striking example of the backwardness of medicine in some departments, and everyone who adds to our knowledge of the pathology and treatment of this affection will be sure of grateful recognition by the profession at large. A large number of cases of angina are not typical cases, and often the medical attendant, by finding out the cause of the anginal attack, may be able to win the eternal gratitude of his patient by enabling the latter, with suitable treatment, successfully to cope with it. It is therefore important to recognise the various forms of anginal attacks, not all of which are as accurately described in the text-books as they might be. One of these is gastric angina pectoris—a hopelessly ridiculous name, but one which, in these days when our profession is much too busy to see the humorous side of its theories and its nomenclature, will serve, inasmuch as it draws attention to the fact that the attack is caused by some stomach derangement. Pawinsky, of Warsaw, has recently devoted some attention to the subject, and although one need not agree with him in taking all cases of angina to be due to gastric trouble and in looking upon angina pectoris as directly due to irritation of the vagal nerve endings, it is beyond doubt that in a large number of cases his conclusions hold good and are therefore worthy of attention.

---

Pawinsky lays special stress, in anginal cases, on the "gastro-iliac symptom group." Thus pyrosis is often an antecedent of the attack, and this acidity differs from the "alimentary pyrosis" associated with gastric lesions in not being influenced by the diet. Another frequent association is dysphagia, and anginal patients frequently complain of sensation of fulness in the epigastric region, extreme dryness of the throat, ptyalism, eructations, and cardialgic pains after taking food. That gastro-intestinal movements often initiate an attack is well known. A careful and systematic examination of all patients is strongly urged by the author, who insists upon the comparative frequency of this type of angina, and who regards it as being usually associated with arterio-sclerosis. In the treatment he attaches importance to regulating the diet and to the employment of iodides, diuretics, and, where necessary, cardiac stimulants. The "gastric anginal" patient ought to take his meals at regular intervals frequently, but in small quantities at a time, and ought to rest after each meal, or at least to refrain from violent physical or mental effort. The author has seen a purely vegetable-milk diet work wonders in such patients in some cases; in others he has seen no good results whatever from such a diet, and has allowed an almost exclusively meat diet with benefit. He therefore considers that there are no definite rules as to dieting, but that proper attention must be paid to the "digestive idiosyncrasies" of anginal patients.

A VERY interesting paper has recently appeared, by Dr. F. A. Watkins, on the treatment of various ailments, such as neurasthenia, chronic arthritis, diabetes mellitus, and gout, by the administration of different combinations of phosphorus, based upon a careful analysis of the acidity and phosphoric content of the urine. It is pointed out that French physicians have employed the method for some years past, although it is little known in this country. The assumption is made that the blood is an acid fluid, and that the acidity of the urine is an index of the state of the blood. This is certainly contrary to physiological teaching, but Dr. Watkins contends that the so-called alkalinity of the blood is due to the presence of bicarbonates, which are actually acid salts. The acidity of the blood, on the other hand, is mainly due to acid phosphates, but their acid reaction to litmus is masked owing to excess of the bicarbonates, which turn red litmus blue. The estimations of acidity and of phosphorus content of the urine are worked out in ratios to the specific gravity. In normal conditions the acid ratio (R.A.) ranges between 4 and 5, and the phosphorus ratio (R.P.) lies between 11 and 11.5. The ratio between R.A. and R.P., known as Joulie's coefficient or the acido-phosphoric ratio, should be about 2.45. If the coefficient is increased there is hyperphosphatia, if decreased, hypophosphatia.

IN disease there is considerable variation in the phosphatic ratio. In neurasthenia, for instance, it is excessive in the early stages, but later when the tissues have been impoverished by large losses of phosphates the R.P. becomes much reduced below normal. In the various morbid conditions in which it is contended some form of phosphorus medication is indicated, the particular phosphorus combination is determined by the analysis of the urine. If the acidity is normal phosphates should be given in a neutral form. Joulie has introduced such a salt under the name of sodium sesquiphosphate. Dr. Watkins, however, favours the organic combinations of phosphorus, and finds a preparation of glycerophosphoric acid and pure casein very suitable. If the acidity of the urine is below normal large doses of phosphoric acid may be given, if sufficiently diluted. In conditions of hyperacidity effervescing phosphate of sodium, together with the mineral waters Vichy, Fachingen, or Vals, are indicated. Dr. Watkins gives details of several cases, with analyses of the urine, which show striking results in the improvement of the patient's condition synchronous with the return of the urine to normal. Phosphoric acid and its compounds have for a long time been regarded as important tonic drugs in the treatment of asthenic and nervous disorders, but physicians have not hitherto formulated any clearly-defined means by which their effects on the metabolism of the body can be estimated. The determination of the kind and amount of phosphorus compounds to be administered by urinary analyses, if based upon rational principles, appears to be a decided advance in scientific therapeutics. It is just this kind of rational therapy and freedom from the old, though often well-tried, empirical methods to which all thoughtful physicians are striving.

THE Bier method of treatment by hyperæmia is sufficiently well known not to require any detailed explanation, although it has probably not yet received the recognition in this country which it deserves. Dr. Le Durey, in a recent communication, gives an interesting *résumé* of the results of his experiences of the method. Briefly, two kinds of hyperæmia must be distinguished: venous hyperæmia, produced by an obstruction to the return circulation or by lowering the atmospheric pressure at a particular point by cupping; and arterial hyperæmia produced by vaso-dilation resulting from the local application of heat or an irritant. Venous stasis is generally obtained by means of an elastic band so adjusted as to produce a local œdema, but without obstructing the circulation too much and causing pain. In order to serve as a guide to the correct degree of pressure Dr. Le Durey employs an elastic band graduated in centimetres, and he finds empirically that the pressure is approximately correct if the limb is bound by a turn of the bandage equal in length to the circumference of the limb less one millimetre for every centimetre of the limb's circumference. The duration of the application varies according to the case from one to twenty-two hours. The maximum dose of twenty-two hours is applicable only to very acute cases. The production of venous stasis by cupping requires less delicacy in adjustment, and the duration of the application is not more than about three-quarters of an hour.

VENOUS hyperæmia is especially applicable to acute inflammatory conditions, and the author's experiences conform to those of Bier, Sick, Strick, and others in the efficacy of the method for such acute local affections as furuncles, abscesses, puerperal mastitis, lymphangitis and phlegmonous conditions. Dr. Le Durey is especially enthusiastic for this method of treatment for all cases of suppurating wounds, infected compound fractures, and severe injuries to a limb. In cases of acute arthritis, apart from the curative effect of the elastic bandage, he finds that two or three applications produce a remarkable diminution in the pain which allows of early passive movements of the joint. Although the method was originally designed for tuberculous joints, the author has obtained less success in this class of case than in other conditions. Arterial hyperæmia is usually produced by the various forms of hot-air bath, and is suitable for chronic conditions, especially rheumatic, of the joints and other parts. Dr. Le Durey finds the results very various in apparently identical cases. In some he finds the pain and other symptoms actually aggravated by the treatment, and he is of opinion that the indications for each of the varieties of hot-air treatment are at present not sufficiently precise. Persistent œdema following fractures is much improved by hot-air baths at 110°-115° for an hour a day, and hæmarthroses and hydrarthroses are similarly benefited after respiration. The author has also obtained some good results with varicose ulcers. He considers that these various forms of the Bier method of treatment are quite free from danger if carried out with proper care and skill.

